# Supplementary material for: Genetic Diversity and Population Structure of Tufted Deer (Elaphodus cephalophus) in Chongqing, China
Source: Animals (Basel). 2025 Jul 31;15(15):2254. doi: 10.3390/ani15152254 (PMC12345541; doi:10.3390/ani15152254)
Supplement: Supplementary file 1 [file animals-15-02254-s001.zip › animals-3720076-supplementary.pdf]

animals

Supplementary Materials

1.Supplementary Figures

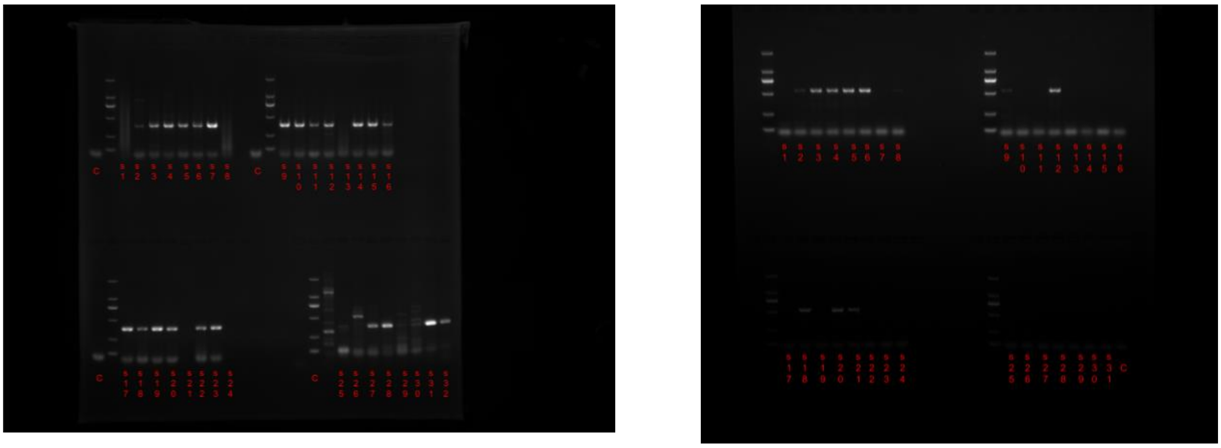

(a) (b)

Figure S1. .PCR electrophoresis results of tufted deer Cyt b gene (a) and D-loop region (b).

Only representative electrophoresis results are shown.

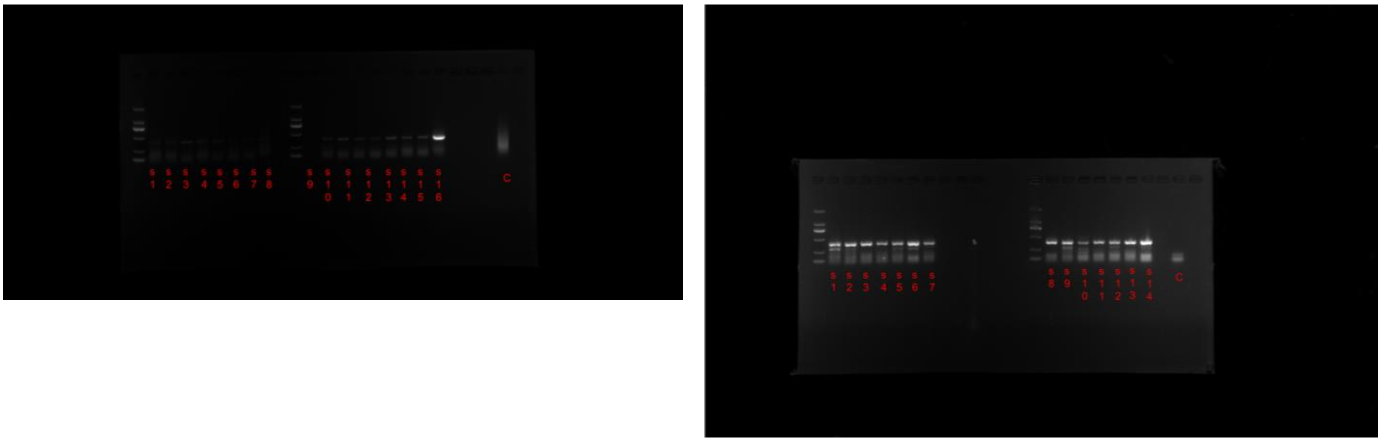

(a) (b)

Figure S2. Electrophoresis results of gel-extracted PCR products from the tufted deer Cyt b gene (a) and D-loop region (b).

Only representative electrophoresis results are shown.

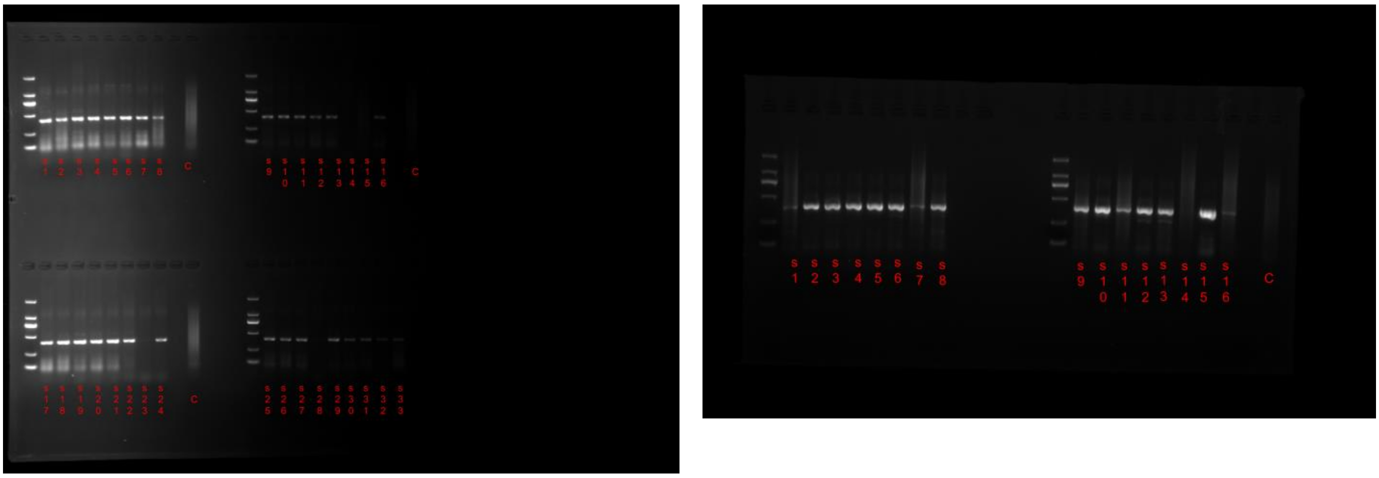

(**a**) (**b**)

Figure S3. Colony PCR electrophoresis results of the tufted deer Cyt b gene (a) and D-loop region (b).

Only representative electrophoresis results are shown.

2.Supplementary Tables

Table S1. Information of reference sequences used in this study

Accession Number	Organism	Name
MN248532	*Elaphodus cephalophus*	Cyt b
MN251783	*Elaphodus cephalophus*	Cyt b
NC_050383	*Elaphodus cephalophus*	Cyt b
DQ379305	*Elaphodus cephalophus*	Cyt b
JN242417	*Elaphodus cephalophus*	Cyt b
JN242418	*Elaphodus cephalophus*	Cyt b
DQ873526	*Elaphodus cephalophus*	Cyt b
MN248532	*Elaphodus cephalophus*	D-loop
MN251783	*Elaphodus cephalophus*	D-loop
DQ873526	*Elaphodus cephalophus*	D-loop
MT726046	*Elaphodus cephalophus*	D-loop
KT152914	*Elaphodus cephalophus*	D-loop
KT152915	*Elaphodus cephalophus*	D-loop
KT152916	*Elaphodus cephalophus*	D-loop
KT152917	*Elaphodus cephalophus*	D-loop
KT152918	*Elaphodus cephalophus*	D-loop
KT152919	*Elaphodus cephalophus*	D-loop
KT152920	*Elaphodus cephalophus*	D-loop
KT152921	*Elaphodus cephalophus*	D-loop
KT152922	*Elaphodus cephalophus*	D-loop
KT152923	*Elaphodus cephalophus*	D-loop
KT152924	*Elaphodus cephalophus*	D-loop
KT152925	*Elaphodus cephalophus*	D-loop
KT152926	*Elaphodus cephalophus*	D-loop
KT152927	*Elaphodus cephalophus*	D-loop
KT152928	*Elaphodus cephalophus*	D-loop
KT152929	*Elaphodus cephalophus*	D-loop

Table S2. Haplotype distribution of tufted deer in the Cyt b gene and D-loop regions

Items	Haplotypes	Individual No.	Number
Cyt b	Hap_1	JF-01、JF-02、JF-03、JF-11	4
	Hap_2	JF-04、JF-05	2
	Hap_3	JF-06、JF-13	2
	Hap_4	JF-07	1
	Hap_5	JF-08、JF-12	2
	Hap_6	JF-09、JF-10	2
	Hap_7	SM-01、SM-21	2
	Hap_8	SM-02、SM-16	2
	Hap_9	SM-03、SM-17	2
	Hap_10	SM-04	1
	Hap_11	SM-05、SM-06、SM-07、SM-09、SM-13、SM-19	6
	Hap_12	SM-08	1
	Hap_13	SM-10、SM-20、NEM-11	3
	Hap_14	SM-11、NEM-08、NEM-10	3
	Hap_15	SM-12	1
	Hap_16	SM-14、NEM-01	2
	Hap_17	SM-15	1
	Hap_18	SM-18	1
	Hap_19	NEM-02	1
	Hap_20	NEM-03	1
	Hap_21	NEM-04、NEM-06	2
	Hap_22	NEM-05	1
	Hap_23	NEM-07	1
	Hap_24	NEM-09	1
	Hap_25	NEM-12	1
D-loop	Hap_1	JF-01、JF-09、SM-10、SM-12	4
	Hap_2	JF-02、SM-20	1
	Hap_3	JF-03	1
	Hap_4	JF-04、JF-06、JF-10、JF-11、SM-15	5
	Hap_5	JF-05	1
	Hap_6	JF-07	1
	Hap_7	JF-08	1
	Hap_8	JF-12	1
	Hap_9	JF-13	1
	Hap_10	SM-01	1
	Hap_11	SM-02	1
	Hap_12	SM-03、NEM-10	2
	Hap_13	SM-04	1

Hap_14	SM-05、SM-07、SM-19	3
Hap_15	SM-06、SM-09	2
Hap_16	SM-08	1
Hap_17	SM-11	1
Hap_18	SM-13	1
Hap_19	SM-14	1
Hap_20	SM-16、SM-21、NEM-01	3
Hap_21	SM-17	1
Hap_22	SM-18	1
Hap_23	NEM-02	1
Hap_24	NEM-03	1
Hap_25	NEM-04、NEM-05、NEM-06	3
Hap_26	NEM-07	1
Hap_27	NEM-08	1
Hap_28	NEM-09	1
Hap_29	NEM-11	1
Hap_30	NEM-12	1
